# Cytotoxic, Anti-Migration, and Anti-Invasion Activities on Breast Cancer Cells of Angucycline Glycosides Isolated from a Marine-Derived *Streptomyces* sp.

**DOI:** 10.3390/md17050277

**Published:** 2019-05-09

**Authors:** Xin-Ying Qu, Jin-Wei Ren, Ai-Hong Peng, Shi-Qi Lin, Dan-Dan Lu, Qian-Qian Du, Ling Liu, Xia Li, Er-Wei Li, Wei-Dong Xie

**Affiliations:** 1 College of Marine Science, Shandong University at Weihai, Weihai 264209, China; quxinying321@163.com (X.-Y.Q.); pengahsdu@163.com (A.-H.P.); lsqsd@outlook.com (S.-Q.L.); 18304019094@163.com (D.-D.L.); 15536791677@163.com (Q.-Q.D.); xiali@sdu.edu.cn (X.L.); 2 State Key Laboratory of Mycology, Institute of Microbiology, Chinese Academy of Sciences, Beijing 100101, China; renjw@im.ac.cn (J.-W.R.); liul@im.ac.cn (L.L.)

**Keywords:** *Streptomyces*, angucycline, saquayamycin, vineomycin, cytotoxicity, migration, breast cancer cell, MDA-MB-231

## Abstract

Four angucycline glycosides were previously characterized from marine-derived *Streptomyces* sp. OC1610.4. Further investigation of this strain cultured on different fermentation media from that used previously resulted in the isolation of two new angucycline glycosides, vineomycins E and F (**1**–**2**), and five known homologues, grincamycin L (**3**), vineomycinone B_2_ (**4**), fridamycin D (**5**), moromycin B (**7**), and saquayamycin B_1_ (**8**). Vineomycin F (**2**) contains an unusual ring-cleavage deoxy sugar. All the angucycline glycosides isolated from *Streptomyces* sp. OC1610.4 were evaluated for their cytotoxic activity against breast cancer cells MCF-7, MDA-MB-231, and BT-474. Moromycin B (**7**), saquayamycin B_1_ (**8**), and saquayamycin B (**9**) displayed potent anti-proliferation against the tested cell lines, with IC_50_ values ranging from 0.16 to 0.67 μM. Saquayamycin B (**9**) inhibited the migration and invasion of MDA-MB-231 cells in a dose-dependent manner, as detected by Transwell and wound-healing assays.

## 1. Introduction

Angucyclines and their aglucones (angucyclinones) are a class of natural products containing an angularly assembled tetracyclic scaffold or a corresponding rearranged frame in the structure. They are the largest family of polycyclic aromatic polyketides produced by Gram-positive actinomycetes [1]. Since the first member of the class, tetrangomycin, was identified from *Streptomyces rimosus* in 1965 [1], dozens of angucyclines and angucyclinones have been found to display prominent cytotoxic and anti-proliferative properties [2,3,4]. Although their severe in vivo toxicity and limited water-solubility restricted their clinical application, this class of natural products still continuously draws attention due to their structural diversity and anti-neoplastic potential [5,6,7,8]. To date, several subclasses of angucycline derivatives have been extensively investigated regarding their in vitro and in vivo anti-tumor effects. For instance, landomycin E displayed promising anti-cancer activity against multidrug-resistant cancer cells and induced apoptotic cell death as a consequence of rapid mitochondrial damage [9]. Further investigation indicated that rapid H_2_O_2_ generation and complex caspase activation contribute to its anti-neoplastic effects [10]. Jadomycins are atypical angucyclinones containing nitrogenous heterocycles in their structure, and jadomycins B and F were reported to induce DNA cleavage through the generation of cytosolic superoxide or the inhibition of type II topoisomerases, leading to the death of multidrug-resistant breast cancer cells [11,12]. Lomaiviticins were first isolated from *Micromonospora lomaivitiensis* as dimers of kinamycin angucyclines with two diazofluorene functional groups [3]. One of them, lomaiviticin A, exhibited remarkable cytotoxicity against a panel of human cancer cells at nanomolar–picomolar concentrations by inducing double-strand breaks in DNA, and it is currently under preclinical evaluation [13,14,15]. Accordingly, angucyclines are still considered as promising candidates for anti-tumor drug development. 

Angucyclines were mainly separated from terrestrial actinomycetes, though in recent years an increasing number of them have been identified from marine actinomycetes associated with seafloor sediments [4,8,16,17,18,19], sponges [7], and mangrove forests [20]. We also initiated a screening for angucycline-producing strains employing PCR amplification of the β-ketoacyl synthase (KSα) gene from marine actinomycetes, and a *Streptomyces* sp. designated as OC1610.4 was obtained from the intertidal sediments. Its 16S rRNA nucleotide sequence (GenBank number: MK045847) is similar to those of *Streptomyces chromofuscus* (FJ486284) and *Streptomyces lannensis* (KM370050), with 81.8% and 81.6% similarity, respectively (Supplementary Figure S1). After this strain was cultured in Morel & Wetmore Modification medium (S-medium), four angucycline glycosides were identified, and one of the isolates, saquayamycin B, displayed potent cytotoxicity on human hepatoma carcinoma cells [21]. For the purpose of discovering more diversified analogues with cytotoxicity, three kinds of medium—Gauze’s synthetic solid medium (GAU), yeast extract–malt extract starch medium (YMS), and yeast extract–malt extract agar medium (YMEA)—were further employed to reculture this strain. Thin-layer chromatography (TLC) profiles showed that more yellow spots displaying orange fluorescence under UV 365 nm light were observed in the EtOAc extract of GAU. Large-scale fermentation using GAU and isolation resulted in the identification of another seven angucyclines including two new derivatives. This paper reports their structure identification and cytotoxic, anti-migration, and anti-invasion activities on breast cancer cells of angucyclines obtained from *Streptomyces* sp. OC1610.4. 

## 2. Results and Discussion

**Figure 1:**
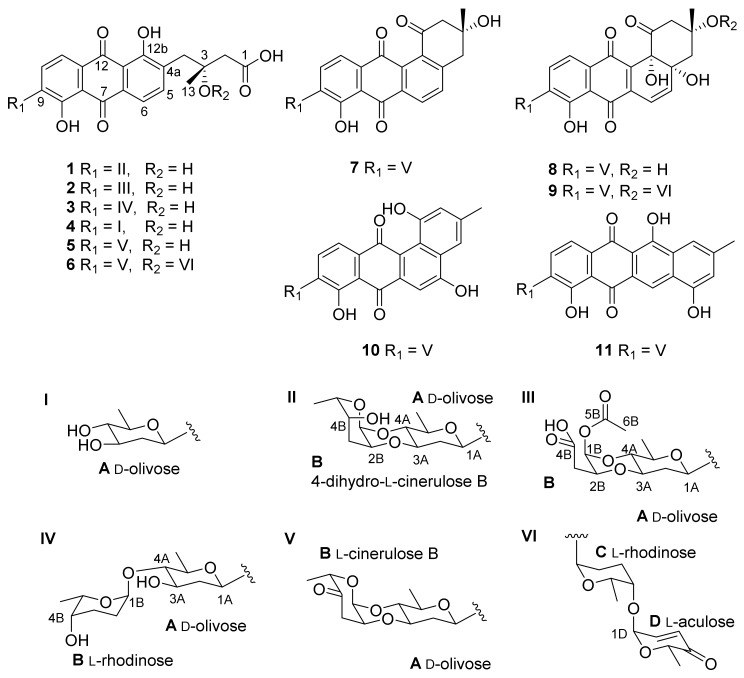
Structures of **1**–**11**.

The strain *Streptomyces* sp. OC1610.4 was previously shaking-cultured in liquid S-medium (10 g/L glucose, 4 g/L yeast extract, 4 g/L K_2_HPO_4_, 2 g/L KH_2_PO_4_, 0.5 g/L MgSO_4_·7H_2_O, and 3.0% sea salt), and four angucycline glycosides—vineomycin D (**6**), saquayamycin B (**9**), landomycin N (**10**), and galtamycin C (**11**)—were characterized from the fermentation broth [21]. After being recultured in solid GAU (20 g/L amylogen, 1 g/L KNO_3_, 0.5 g/L NaCl, 0.5 g/L K_2_HPO_4_·H_2_O, 0.5 g/L MgSO_4_·H_2_O, and 0.01 g/L FeSO_4_·H_2_O), two new rearranged angucyline glycosides, vineomycins E and F (**1** and **2**), along with five known homologues, grincamycin L (**3**) [19], vineomycinone B_2_ (**4**) [22], fridamycin D (**5**) [23], moromycin B (**7**) [24], and saquayamycin B_1_ (**8**) [6,25], were identified (Figure 1). 

**Figure 2:**
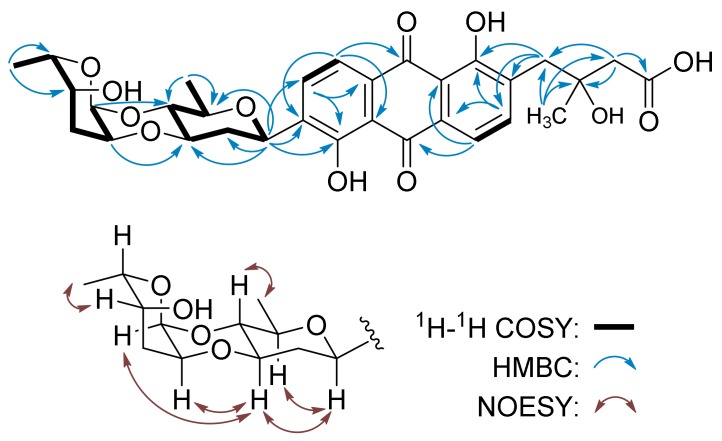
COSY, HMBC, and key NOESY correlations for **1**.

Vineomycin E (**1**) was isolated as a trace constituent. Its molecular formula (C_31_H_34_O_12_) was determined by the *m/z* 599.2122 ([M + H]^+^, calc.d for C_31_H_3_5O_12_, 599.2129) from high-resolution electrospray ionization mass spectrometry (HR-ESI-MS). The ^1^H NMR of **1** showed the signals of two pairs of ortho-coupled aromatic protons at δ_H_ 7.68 (m, H-6) and 7.70 (m, H-5), and δ_H_ 7.72 (d, J = 7.3 Hz, H-11) and 7.84 (d, J = 7.3 Hz, H-10), characteristic of rearranged tricyclic angucyclines, e.g., grincamycin L (**3**) [19], vineomycinone B_2_ (**4**) [22], and fridamycin D (**5**) [23]. The oxygenated methine proton signals at δ_H_ 4.92 (brd, *J* = 11.4 Hz, H-1A) and 4.97 (brs, H-1B), corresponding carbon signals at δ_C_ 72.2 (C-1A) and 91.0 (C-1B) ppm assigned by HMQC correlations, along with the two doublets of methyl groups at δ_H_ 1.26 (d, *J* = 6.9 Hz, H-6B) and 1.31 (d, *J* = 5.8 Hz, H-6A), implied the presence of two deoxy sugars, one of which formed a C-glycoside due to its anomeric carbon resonating at δ_H_ 72.2 [1]. The ^13^C NMR signals of **1** were very similar to those of fridamycin D (**5**) [23], except for the presence of a signal at δ_C_ 64.1 (C-4B) and the absence of a signal above δ_C_ 200, which indicated that **1** and fridamycin D (**5**) differed in the deoxy sugars. In the HMBC spectrum of **1**, the correlations from H-5 (δ_H_ 7.70) to C-6a (δ_C_ 132.9) and C-12b (δ_C_ 162.4); H-6 (δ_H_ 7.68) to C-7 (δ_C_ 189.2), C-12a (δ_C_ 116.6) and C-4a (δ_C_ 136.3); H-10 (δ_H_ 7.84) to C-8 (δ_C_ 159.7) and C-11a (δ_C_ 133.3); and H-11 (δ_H_ 7.72) to C-7a (δ_C_ 116.6), C-9 (δ_C_ 139.2), and C-12 (δ_C_ 189.3) supported the anthraquinone skeleton and the two hydroxyl groups at C-9 and C-12b (Figure 2). The side chain and its location at C-4a were deduced by the HMBC correlations from H-13 (δ_H_ 1.27) to C-2 (δ_C_ 46.7), C-3 (δ_C_ 73.0) and C-4 (δ_C_ 41.2); H-2 (δ_H_ 2.48, 2.51) to C-1 (δ_C_ 176.4) and C-4 (δ_C_ 41.2); and H-4 (δ_H_ 3.00, 3.09) to C-4a (δ**_C_** 136.3), C-5 (δ_C_ 140.8), and C-12b (δ_C_ 162.4). Tricyclic angucyclines are generally derived from typical angucyclines with the same tetracyclic core structure under acidic conditions [1]; therefore, the absolute configuration of C-3 is proposed to be the same as that of saquayamycin B (**9**) and other tricyclic angucyclines, e.g., grincamycin L (**3**) and grincamycin B [19,26]. The two deoxy sugars were deduced to be D-olivose and 4-dihydro-l-cinerulose B by the COSY correlations from H-1A (*δ*_H_ 4.92) through H-6A (*δ*_H_ 1.31), and correlations from H-1B (*δ*_H_ 4.97) through H-6B (*δ*_H_ 1.26) (Figure 2). The HMBC correlations from H-1B (*δ*_H_ 4.97) to C-4A (*δ*_C_ 75.1), and H-2B (*δ*_H_ 3.88) to C-3A (*δ*_C_ 78.0) suggested that they constitute a 4-dihydro-l-cinerulose B-(1→4,2→3)-olivosyl group (Figure 2). The NOESY correlations of H-1/H-3A,5A and H4A/H-6A confirmed the relative configurations of the *β*-D-olivosyl group (Figure 2). The simultaneously appearing NOESY correlations H-3A/H-1B and H-3A/H-2B suggested that H-1B and H-2B are located in the axial and equatorial directions, respectively. The hydroxyl group at C-4B in the 4-dihydro-α-L-cinerulose B moiety was assigned in the axial direction by the NOESY correlation H-6B/H-4B. Thus, the structure of **1** was established and named vineomycin E (**1**). Deoxy sugar 4-dihydro-L-cinerulose B rarely occurred in the structure of an angucycline, and only one example, namely PI-083, has been reported to comprise a 4-dihydro-L-cinerulose B group to date [27].

**Figure 3:**
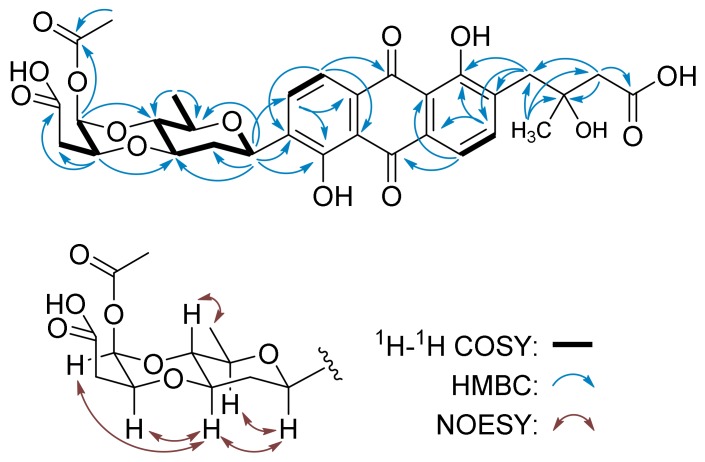
COSY, HMBC, and key NOESY correlations for **2**.

Vineomycin F (**2**) was isolated as a yellow solid and has the molecular formula C_31_H_32_O_14_, determined by the *m/z* 629.1867 ([M + H]^+^, calc.d for C_31_H_33_O_14_, 629.1870) from HR-ESI-MS. The ^1^H NMR spectrum of **2** displayed the resonances of four aromatic protons and a set of aliphatic protons, similar to those of **1**. The prominent difference is that the anomeric proton signal of the terminal deoxy sugar in **2**, whose corresponding carbon resonated at δ_C_ 90.5 assigned by the HMQC spectrum, shifted to downfield *δ*_H_ 6.06 (H-1B). The ^13^C NMR spectrum of **2** showed 31 carbon signals in which δ_C_ 169.8, 171.4, and 173.8 were assigned to three carboxyl or ester carbonyl carbons. The chemical shift of the methyl group at *δ*_H_ 2.17 (s, H-6B) and the HMBC correlation from this signal to carbon at *δ*_H_ 169.8 (C-5B) supported the presence of an acetyl group (Figure 3). After the signals of protons and carbons in **2** were completely assigned by HMQC and COSY spectra (Table 1), the structure of aglycone was established to be identical to that of **1** by the HMBC correlations associated with the four aromatic protons (*δ*_H_ 7.78, 7.84, 7.85, and 7.96) and the methane protons (*δ*_H_ 2.56, 2.59, and 3.10) (Figure 3). The presence of the D-olivose moiety, including its configurations, was confirmed by the COSY correlations from H-1A (δ_H_ 5.03, d, *J* = 9.1 Hz) through H-6A (*δ*_H_ 1.26, d *J* = 5.6 Hz) and the NOESY correlations H-1/H-3A,5A and H4A/H-6A. The HMBC correlations from H-1A (*δ*_H_ 5.03) to C-8 (δ_C_ 159.6), C-9 (δ_C_ 138.8), and C-10 (δ_C_ 134.2) indicated the attachment of *β*-D-olivose at C-8 to form a C-glycoside (Figure 3). The COSY correlations from H-1B (*δ*_H_ 6.06) through H-3B (*δ*_H_ 2.54), together with the HMBC correlations from H-2B (*δ*_H_ 4.34) to the carboxyl carbon at *δ*_H_ 171.4 (C-4B), suggested that the terminal sugar is a butyrate acid derivative. In addition, it linked with *β*-D-olivose through two ether bonds, which were deduced by the HMBC correlations from H-1B (δ_H_ 6.06) to C-4A (*δ*_C_ 75.3) and H-2B (δ_H_ 4.34) to C-3A (*δ*_C_ 74.6). The downfield shift of H-1B to δ_H_ 6.06 and the HMBC correlation from H-1B to the carbonyl carbon at *δ*_C_ 169.8 (C-5B) implied that the acetyl group attached at C-1B. The acetyl group and the butyrate acid derivative were probably the results of an oxidative break of the C–C bond between C-4 and C-5 in L-cinerulose B (Figure 3).

**Figure 4:**
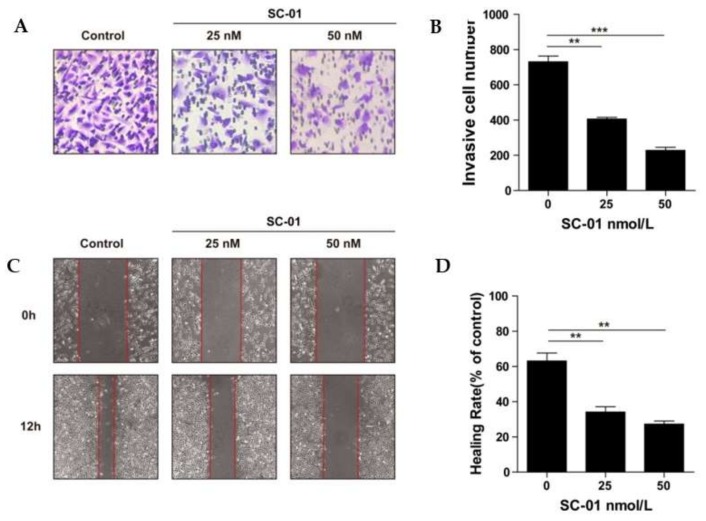
Saquayamycin B (**9**) treatment dose-dependently inhibited invasion and migration in the breast cancer cell line MDA-MB-231. Cell invasion and migration were observed with incubation for 12 h by Transwell and wound-healing assays. **A**: cresyl violet staining in the Transwell assay, captured by a microscope (100× magnification). **B**: quantification by counting the number of cells in the Transwell assay. **C**: effects of wound-healing captured by a microscope (100× magnification). **D**: analysis of the wound-healing rate. Results are presented as mean ± SD. ** *p* < 0.01, *** *p* < 0.001 compared to the control group.

Breast cancer is among the most common types of cancer affecting women worldwide and is the leading cause of cancer death in women [28]. The migration and invasion of breast cancer cells allow the cells to enter lymphatic vessels or the bloodstream and lead to cancer deterioration, relapse, difficult eradication, and even incurability [29]. Several investigations have reported that some angucyclines, such as jadomycins B, S, and F, are capable of inducing apoptosis in drug-resistant breast cancer cells [11,12]. Moromycin B (**7**) was also reported to show significant cytotoxicity against MCF-7 human breast cancer cells, with a GI_50_ value of 5.6 μM [24]. Thus, we evaluated the cytotoxic activity of isolated angucyclines on the MCF-7, MDA-MB-231, and BT-474 cell lines employing the 3-(4,5-dimethylthiazol-2-yl)-2,5-diphenyltetrazolium bromide (MTT) method (Table 2). Among the tested compounds, moromycin B (**7**), saquayamycin B_1_ (**8**), and saquayamycin B (**9**) displayed remarkable cytotoxicity against breast cancer cells, with IC_50_ values ranging from 0.16 to 0.67 μM. Vineomycin E (**1**) and fridamycin D (**5**) displayed medium cytotoxicity on the tested cells. After treatment of the aggressive triple-negative cell line MDA-MB-231 with saquayamycin B (**9**) at concentrations of 25 and 50 nM for 12 h, the invasion and migration capabilities of MDA-MB-231 cells were found to be depressed by Transwell and wound-healing assays (Figure 4). 

## 3. Materials and Methods 

### 3.1. General Experimental Procedures

Silica gel (200–300 mesh), used in column chromatography (CC), and silica gel GF_254_ (10–40 µm), used in thin layer chromatography (TLC), were purchased from Qingdao Marine Chemical Factory in China. Optical rotations were recorded with an Anton Paar MCP 200 polarimeter with a sodium lamp (589 nm) (Anton Paar GmbH, Graz, Austria). UV spectra were measured on a Genesys 10S UV-Vis spectrometer (Thermo Fisher Scientific Ltd., Waltham, MA, USA); IR spectra were recorded with a Nicolet IS5 FT-IR spectrometer (Thermo Fisher Scientific, Waltham, MA, USA); 1D and 2D NMR spectra were recorded on a Bruker AVANCE III 500 spectrometer (Bruker Inc., Karlsruhe, Germany). HPLC-HR-ESI-MS was performed on an Agilent 1200HPLC/6520QTOFMS (Agilent Technologies Inc., Santa Clara, CA, USA). Semipreparative HPLC isolation was conducted on an Agilent 1260 Infinity II (Agilent Technologies Inc., Santa Clara, CA, USA) with an ODS column (10 × 250 mm, YMC-Triart C18, YMC Co. Ltd., Tokyo, Japan). 

### 3.2. Actinomycetes Strain 

The strain OC1610.4 was obtained from intertidal sediment using the method reported in our previous research [19]. DNA extraction and PCR amplification of 16S rRNA were conducted according to the instructions of the DNA isolation kit and PCR kit supplied by Shanghai Sangon Biotech Co., China, and 16S rRNA was sequenced by the same company. The 16S rRNA sequence was deposited at GenBank (accession no. MK045847), and the closely related taxa were retrieved from GenBank using BLAST software. The voucher strain (no. OC1610.4) was deposited at the Laboratory of Natural Products Chemistry, College of Marine Science, Shandong University at Weihai. 

### 3.3. Fermentation, Extraction, and Isolation

The strain OC1610.4 was first picked out from the deposit tube and inoculated in 3 Erlenmeyer flasks (500 mL), each of which contained 100 mL GAU. After being shaking-cultured at 140 rpm, at 28 °C for 3 days, the spore and mycelia suspension was plated on Petri dishes containing 40 L GAU (20–30 mL/dish) and cultured for 9 days at 32 °C. The fermentation medium was sheared to pieces (<2 × 2 cm) and extracted with EtOAc 4 times to give 8.6 g crude extract. The extract was subjected to silica gel CC (260 g, 200–300 mesh) eluting with *n*-hexane-acetone (10:1, 5:1, 2:1 and acetone). According to the TLC spots displaying orange fluorescence under UV 365 nm light, one fraction (F_1_) was obtained from the n-hexane-acetone (5:1) eluent, and 4 fractions (F_2a_–F_2d_) were obtained from the *n*-hexane-acetone (2:1) eluent. Fraction F_1_ (320 mg) was purified by semipreparative HPLC eluting with H_2_O (0.1% CH_3_CO_2_H)-CH_3_OH (80:20, *v/v*) to give 3-(3-hydroxy-phenyl)-propionic acid (5.6 mg, 14.2 min). Fraction F_2_a (267 mg) was isolated by semipreparative HPLC eluting with H_2_O (0.1% CH_3_CO_2_H)-CH_3_OH (80:20, *v/v*) to give **7** (3 mg, *t*_R_ 34.0 min) and **5** (9 mg, *t*_R_ 30.8 min). Fraction F_2b_ (175 mg) was isolated by semipreparative HPLC eluting with H_2_O (0.1% CH_3_CO_2_H)-CH_3_OH (80:20, *v/v*) to give **8** (4 mg, *t*_R_ 24.5 min). Fraction F_2c_ (109 mg) was isolated by semipreparative HPLC eluting with H_2_O (0.1% CH_3_CO_2_H)-CH_3_OH (83:17, *v/v*) to give **2** (8 mg, *t*_R_ 14.5 min), **1** (2 mg, *t*_R_ 16.7 min), and **3** (2 mg, *t*_R_ 18.2 min). F_2d_ (119 mg) was purified by semipreparative HPLC eluting with H_2_O (0.1% CH_3_CO_2_H)-CH_3_OH (67:33, *v/v*) to give **4** (9 mg, *t*_R_ 45.6 min). 

*Vineomycin E* (**1**): yellow amorphous powder; ${\left[\alpha \right]}_{25}^{D}$ +28.4 (*c* 0.20, CH_2_Cl_2_); UV (MeOH) λ_max_ (log ε) 229 (3.53), 257 (3.37), 294 (2.97) nm; IR (neat) *ν*_max_ 3402, 2977, 2928, 2853, 1719, 1631, 1435, 1373, 1259, 1073, 767, 751 cm^–1^; ^1^H NMR (500 MHz, CD_3_OD) and ^13^C NMR (125 MHz, CD_3_OD) data, see Table 1; HR-ESI-MS *m/z* 599.2122 ([M + H]^+^, calc.d for C_31_H_35_O_12_, 599.2129).

*Vineomycin F* (**2**): yellow amorphous powder; ${\left[\alpha \right]}_{25}^{D}$ +23.2 (*c* 0.50, CH_2_Cl_2_); UV (MeOH) λ_max_ (log ε) 229 (3.11), 257 (2.95), 294 (2.55) nm; IR (neat) *ν*_max_ 3393, 2966, 2927, 2855, 1717, 1627, 1607, 1433, 1369, 1260, 1074, 950, 798 cm^–1^; ^1^H NMR (500 MHz, acetone-d_6_) and ^13^C NMR (125 MHz, acetone-d_6_) data, see Table 1; HR-ESI-MS *m/z* 629.1867 ([M + H]^+^, calc.d for C_31_H_33_O_14_, 629.1870).

### 3.4. Cell Culture

Human breast cancer cells MCF-7, MDA-MB-231, and BT-474 were bought from the Shanghai Institute for Biological Sciences, Chinese Academy of Sciences, China. The cell lines were cultured in RPMI-1640 medium (Hyclone) containing 10% fetal bovine serum (FBS) supplemented with 100 units/mL of penicillin and 100 μg/mL of streptomycin. All cells were incubated in 5% CO_2_ at 37 °C.

### 3.5. MTT Assay

The cytotoxic activity of compounds **1**–**11** against breast cancer cells MCF-7, MDA-MB-231, and BT-474 was determined using the 3-(4,5-dimethylthiazole-2-yl)-2,5-diphenyltetrazolium bromide (MTT) assay as described in our previous research [30]. Doxorubicin was used as a positive control drug and deionized H_2_O with the same DMSO concentration was used as a parallel control. 

### 3.6. Wound-Healing Assay

The anti-migration effect of saquayamycin B (**9**) against breast cancer cells MDA-MB-231 was evaluated using a wound-healing assay [31]. Briefly, MDA-MB-231 cells were cultured in a 24-well plate at a concentration of 5 × 10^5^ cells/mL with RPMI-1640 (10% FBS). When the cell density grew to 90%, linear gaps were scratched by micropipette tips, and the suspension cells were flushed out using phosphate-buffered saline (PBS). Then, the cells in the plate were starved for 12 h to eliminate the interference of proliferation. Wound healing rate (%) = [1 – (scratch width of saquayamycin B-treated group/scratch width of control group)] × 100%. 

### 3.7. Transwell Assay

A Transwell assay was employed to evaluate the invasion capacity of MDA-MB-231 cells [29]. A mixture of Matrigel and serum-free RPMI-1640 (1:8) was precoated on the upper chamber membrane of the filter insert. Then, 200 μL cell suspension (5 × 10^5^ cells/mL) in serum-free RPMI-1640 was poured into the upper chamber and treated with 25 and 50 nM saquayamycin B or DMSO for 12 h. RPMI-1640 medium containing 20% FBS was filled in the lower chamber as a chemoattractant. The invading cells in the lower chamber were fixed with methanol for 15 min and stained by 0.5% cresyl violet, followed by washing 3 times with double-distilled water and air drying. Three random views were photographed, and the invasive cells were counted under a Nikon TE2000 microscope with NIS elements viewer 4.2.0 software (Nikon Instech Co., Ltd., Tokyo, Japan).

## 4. Conclusions

In total, 11 angucycline glycosides, including two new compounds, vineomycin E (**1**) and vineomycin F (**2**), were identified from the fermentation medium of marine-derived *Streptomyces* sp. OC1610.4. Among the isolated angucycline glycosides, vineomycin E (**1**) contains a rarely occurring deoxy sugar, 4-dihydro-l-cinerulose, and vineomycin F (**2**) contains an unusual ring-cleavage deoxy sugar. Moromycin B (**7**), saquayamycin B_1_ (**8**), and saquayamycin B (**9**) displayed potent cytotoxic activity against breast cancer cells MCF-7, MDA-MB-231, and BT-474, with IC_50_ values ranging from 0.16 to 0.67 μM. Saquayamycin B (**9**) inhibited the migration and invasion of MDA-MB-231 cells in a dose-dependent manner.

## Figures and Tables

**Table 1:** ^1^H and ^13^C NMR data of **1** and **2** (500 MHz and 125 MHz) ^a^.

No.	1 ^b^		2 ^c^	
	*δ*_C_	*δ*_H_	*δ*_C_	*δ*_H_
1	176.4 s	-	173.8 s	-
2	46.7 t	2.48, d (15.5) 2.51, d (15.5)	45.5 t	2.56, d (15.2) 2.59, d (15.2)
3	73.0 s	-	72.1 s	-
4	41.2 t	3.01, d (13.1) 3.08, d (13.1)	40.8 t	3.10, brs
4a	136.3 s	-	136.2 s	-
5	140.8 d	7.70, m^c^	140.8	7.85, d (5.6)
6	119.6 d	7.68, m^c^	119.3 d	7.78, d (5.6)
6a	132.9 s	-	132.5 s	-
7	189.2 s	-	189.1 s	-
7a	116.6 s	-	116.3 s	-
8	159.7 s	-	159.6 s	-
9	139.2 s	-	138.8 s	-
10	134.4 d	7.84, d (7.3)	134.2 d	7.96, d (6.6)
11	120.1 d	7.72, d (7.3)	119.8 d	7.84, d (6.6)
11a	133.3 s	-	133.0 s	-
12	189.3 s	-	189.2 s	-
12a	116.6 s	-	116.4 s	-
12b	162.4 s	-	162.3 s	-
13	27.1 q	1.27, s	27.3 q	1.31, s
Sugar A, β-d-olivose				
1A	72.7 d	4.92, brd (11.4)	72.2 d	5.03, brd (9.1)
2A	37.9 t	1.50, m2.38, m	37.3 t	1.56, m2.43, m
3A	78.0 d	3.68, m	77.5 d	3.89, m
4A	75.1 d	3.47, dd (9.3, 9.3)	75.3 d	3.46, dd (7.6, 7.8)
5A	75.6 d	3.56, m	74.6 d	3.67, m
6A	17.9 q	1.31, d (5.8)	17.6 q	1.26, d (5.6)
Sugar B				
1B	91.0 s	4.97, brs	90.5 d	6.06, brs
2B	73.7 d	3.88, m	74.4 d	4.34, m
3B	32.6 t	1.92, m1.96, m	35.9 d	2.54, m
4B	64.1 d	4.12, m	171.4 s	-
5B	74.7 d	4.24, m	169.8 s	-
6B	11.5 q	1.26, d (6.9)	20.8 q	2.17, s

^a^ Residual signals of the solvent as a reference. ^b^ Measured in CD_3_OD. ^c^ Measured in acetone-d_6_.

**Table 2:** Cytotoxicity of **1**–**11** against MCF-7, MDA-MB-231, and BT-474 cells (IC_50_, μM).

Compounds	Cell lines		
	MCF-7	MDA-MB-231	BT-474
**1**	6.07 ± 0.09	7.72 ± 0.76	4.27 ± 2.09
**2**	>20	>20	>20
**3**	>20	>20	>20
**4**	>20	>20	>20
**5**	7.58 ± 1.19	8.01 ± 0.55	6.46 ± 1.92
**6**	>20	>20	>20
**7**	0.42 ± 0.03	0.35 ± 0.03	0.67 ± 0.09
**8**	0.24 ± 0.01	0.16 ± 0.02	0.28 ± 0.09
**9**	0.40 ± 0.01	0.38 ± 0.04	0.41 ± 0.15
**10**	>20	>20	>20
**11**	>20	>20	>20
**Doxorubicin**	0.86 ± 0.64	1.30 ± 0.25	0.39 ± 0.06

## Supplementary Materials

The following are available online at https://www.mdpi.com/1660-3397/17/5/277/s1. This section includes the colonial morphology, 16S rRNA gene sequences data and phylogenetic analysis of *Streptomyces* sp. N1510.2, and the HR-ESI-MS, 1D and 2D NMR spectra for **1**–**2**.

## References

[1] Rohr J., Thiericke R. (1992). Angucycline group antibiotics. Nat. Prod. Rep..

[2] Kharel M.K., Pahari P., Shepherd M.D., Tibrewal N., Nybo S.E., Shaaban K.A., Rohr J. (2012). Angucyclines: Biosynthesis, mode-of-action, new natural products, and synthesis. Nat. Prod. Rep..

[3] He H.Y., Ding W.D., Bernan V.S., Richardson A.D., Ireland C.M., Greenstein M., Ellestad G.A., Carter G.T. (2001). Lomaiviticins A and B, potent antitumor antibiotics from Micromonospora lomaivitiensis. J. Am. Chem. Soc..

[4] Zhang W.J., Liu Z., Li S.M., Lu Y.Z., Chen Y.C., Zhang H.B., Zhang G.T., Zhu Y.G., Zhang G.Y., Zhang W.M. (2012). Fluostatins I–K from the south China sea-derived *Micromonospora rosaria* SCSIO N160. J. Nat. Prod..

[5] Wu C.S., van der Heul H.U., Melnik A.V., Lubben J., Dorrestein P.C., Minnaard A.J., Choi Y.H., van Wezel G.P. (2019). Lugdunomycin, an angucycline-derived molecule with unprecedented chemical architecture. Angew. Chem. Int. Edit..

[6] Shaaban K.A., Ahmed T.A., Leggas M., Rohr J. (2012). Saquayamycins G-K, cytotoxic angucyclines from *Streptomyces* sp. including two analogues bearing the aminosugar rednose. J. Nat. Prod..

[7] Vicente J., Stewart A.K., van Wagoner R.M., Elliott E., Bourdelais A.J., Wright J.L.C. (2015). Monacyclinones, new angucyclinone metabolites isolated from *Streptomyces* sp. M7_15 associated with the Puerto Rican sponge *Scopalina ruetzleri*. Mar. Drugs.

[8] Zhu X.C., Duan Y.W., Cui Z.M., Wang Z., Li Z.X., Zhang Y., Ju J.H., Huang H.B. (2017). Cytotoxic rearranged angucycline glycosides from deep sea-derived *Streptomyces lusitanus* SCSIO LR32. J. Antibiot..

[9] Korynevska A., Heffeter P., Matselyukh B., Elbling L., Micksche M., Stoika R., Berger W. (2007). Mechanisms underlying the anticancer activities of the angucycline landomycin E. Biochem Pharmacol..

[10] Panchuk R.R., Lehka L.V., Terenzi A., Matselyukh B.P., Rohr J., Jha A.K., Downey T., Kril I.J., Herbacek I., van Schoonhoven S. (2017). Rapid generation of hydrogen peroxide contributes to the complex cell death induction by the angucycline antibiotic landomycin E. Free Radical Bio. Med..

[11] Hall S.R., Blundon H.L., Ladda M.A., Robertson A.W., Martinez-Farina C.F., Jakeman D.L., Goralski K.B. (2015). Jadomycin breast cancer cytotoxicity is mediated by a copper-dependent, reactive oxygen species-inducing mechanism. Pharmacol. Res. Perspect..

[12] Hall S.R., Toulany J., Bennett L.G., Martinez-Farina C.F., Robertson A.W., Jakeman D.L., Goralski K.B. (2017). Jadomycins inhibit type II topoisomerases and promote DNA damage and apoptosis in multidrug-resistant triple-negative breast cancer cells. J. Pharmacol. Exp. Ther..

[13] Woo C.M., Beizer N.E., Janso J.E., Herzon S.B. (2014). The cytotoxicity of (-)-lomaiviticin A arises from induction of double-strand breaks in DNA. Nat. Chem..

[14] Colis L.C., Hegan D.C., Kaneko M., Glazer P.M., Herzon S.B. (2015). Mechanism of action studies of lomaiviticin A and the monomeric lomaiviticin aglycon. Selective and potent activity toward DNA double-strand break repair-deficient cell lines. J. Am. Chem. Soc..

[15] Herzon S.B. (2017). The mechanism of action of (-)-lomaiviticin A. Accounts Chem. Res..

[16] Guo L., Xie Z.P., Yang Q., Feng L.L., Zhang L., Zhang Y.Z., Li X.N., Pescitelli G., Zhang S.M. (2018). Kiamycins B and C, unusual bridged angucyclinones from a marine sediment-derived *Streptomyces* sp.. Tetrahedron Lett..

[17] Jin J., Yang X.Y., Liu T., Xiao H., Wang G.Y., Zhou M.J., Liu F.W., Zhang Y.T., Liu D., Chen M.H. (2018). Fluostatins M-Q featuring a 6-5-6-6 ring skeleton and high oxidized A-rings from marine *Streptomyces* sp. PKU-MA00045. Mar. Drugs.

[18] Hu Z.J., Qin L.L., Wang Q.Q., Ding W.J., Chen Z., Ma Z.J. (2016). Angucycline antibiotics and its derivatives from marine-derived actinomycete *Streptomyces* sp. A6H. Nat. Prod. Res..

[19] Yang L., Hou L.K., Li H.Y., Li W.L. (2019). Antibiotic angucycline derivatives from the deepsea-derived *Streptomyces lusitanus*. Nat. Prod. Res..

[20] Gui C., Liu Y.N., Zhou Z.B., Zhang S.W., Hu Y.F., Gu Y.C., Huang H.B., Ju J.H. (2018). Angucycline glycosides from mangrove-derived *Streptomyces diastaticus* subsp. SCSIO GJ056. Mar. Drugs.

[21] Peng A.H., Qu X.Y., Liu F.Y., Li X., Li E.W., Xie W.D. (2018). Angucycline glycosides from an intertidal sediments strain *Streptomyces* sp. and their cytotoxic activity against hepatoma carcinoma cells. Mar. Drugs.

[22] Danishefsky S.J., Biing J.U., Quallich G. (1984). Total synthesis of vineomycin-B_2_ aglycon. J. Am. Chem. Soc..

[23] Maskey R.P., Helmke E., Laatsch H. (2003). Himalomycin A and B: Isolation and structure elucidation of new fridamycin type antibiotics from a marine *Streptomyces* isolate. J. Antibiot..

[24] Abdelfattah M.S., Kharel M.K., Hitron J.A., Baig I., Rohr J. (2008). Moromycins A and B, isolation and structure elucidation of C-glycosylangucycline-type antibiotics from *Streptomyces* sp. KY002. J. Nat. Prod..

[25] Uchida T., Imoto M., Watanabe Y., Miura K., Dobashi T., Matsuda N., Sawa T., Naganawa H., Hamada M., Takeuchi T. (1985). Saquayamycins, new aquayamycin-group antibiotics. J. Antibiot..

[26] Huang H.B., Yang T.T., Ren X.M., Liu J., Song Y.X., Sun A.J., Ma J.Y., Wang B., Zhang Y., Huang C.G. (2012). Cytotoxic angucycline class glycosides from the deep sea actinomycete *Streptomyces lusitanus* SCSIO LR32. J. Nat. Prod..

[27] Kawashima A., Yoshimura Y., Goto J., Nakaike S., Mizutani T., Hanada K., Omura S. (1988). Pi-083, a new platelet-aggregation inhibitor. J. Antibiot..

[28] Lafourcade A., His M., Baglietto L., Boutron-Ruault M.C., Dossus L., Rondeau V. (2018). Factors associated with breast cancer recurrences or mortality and dynamic prediction of death using history of cancer recurrences: The French E3N cohort. Bmc Cancer.

[29] Narod S.A., Sopik V. (2018). Is invasion a necessary step for metastases in breast cancer?. Breast Cancer Res. Tr..

[30] Zheng B.B., Wu L.H., Ma L.S., Liu S.S., Li L., Xie W.D., Li X. (2013). Telekin induces apoptosis associated with the mitochondria-mediated pathway in human hepatocellular carcinoma cells. Biol. Pharm. Bull..

[31] Ma J.H., Qi J., Liu FY., Lin S.Q., Zhang C.Y., Xie W.D., Zhang H.Y., Li X. (2018). Ivalin inhibits proliferation, migration and invasion by suppressing epithelial mesenchymal transition in breast cancer cells. Nutr. Cancer.

