# Supplementary material for: Cytotoxic, Anti-Migration, and Anti-Invasion Activities on Breast Cancer Cells of Angucycline Glycosides Isolated from a Marine-Derived Streptomyces sp
Source: Mar Drugs. 2019 May 9;17(5):277. doi: 10.3390/md17050277 (PMC6562490; doi:10.3390/md17050277)

# Supplementary Materials

# Cytotoxic, Anti-Migration and Anti-Invasion Activities on Breast Cancer Cells of Angucycline Glycosides Isolated from a Marine-Derived *Streptomyces* sp.

**Xin-Ying Qu[1,#], Jin-Wei Ren[2,#], Ai-Hong Peng[1,2,#], Shi-Qi Lin[1], Dan-Dan Lu[1,2], Qian-Qian Du[1,2], Ling Liu[2], Xia Li[1], Er-Wei Li[2,*] and Wei-Dong Xie[1,*]**

[1] College of Marine Science, Shandong University at Weihai, Weihai 264209, China; quxinying321@163.com (X-Y.Q.); pengahsdu@163.com (A-H.P.); lsqsd@outlook.com (S-Q.L.); 18304019094@163.com (D-D.L.); 15536791677@163.com (Q-Q.D.); xiali@sdu.edu.cn (X.L.)

[2] State Key Laboratory of Mycology, Institute of Microbiology, Chinese Academy of Sciences, Beijing 100101, China; renjw@im.ac.cn (J-W.R.); liul@im.ac.cn (L.L.)

[#] These authors contributed equally to this paper.

* Correspondence: liew@im.ac.cn (E-W.L.); wdxie@sdu.edu.cn (W-D.X.); Tel.: +86-10-6480-6141 (E-W.L.); +86-631-568-8303 (W-D.X.)

**Keywords:** *Streptomyces*; angucycline; saquayamycin; vineomycin; cytotoxicity; migration; breast cancer cell; MDA-MB-231

# Contents

**Figure S1.** (**A**) Colony morphology of *Streptomyces* sp. OC1610.4. (**B**) 16S rRNA gene sequence of *Streptomyces* sp. OC1610.4 (GenBank number: MK045847). (**C**) Neighbor-joining phylogenetic tree based on 16S rRNA gene sequences of *Streptomyces* sp. OC1610.4.

**Figure S2**. HR-ESI-MS of **1**.

**Figure S3**. $^{1}$H-NMR spectrum (500 MHz, $CD_3OD$) of **1**.

**Figure S4**. $^{13}$C-NMR spectrum (125 MHz, $CD_3OD$) of **1**.

**Figure S5**. HMQC spectrum (500 MHz, $CD_3OD$) of **1**.

**Figure S6**. HMBC spectrum (500 MHz, $CD_3OD$) of **1**.

**Figure S7**. $^{1}$H-$^{1}$H COSY spectrum (500 MHz, $CD_3OD$) of **1**.

**Figure S8**. NOESY spectrum (500 MHz, $CD_3OD$) of **1**.

**Figure S9**. HR-ESI-MS of **2**.

**Figure S10**. $^{1}$H-NMR spectrum (500 MHz, acetone-$d_6$) of **2**.

**Figure S11**. $^{13}$C-NMR spectrum (125 MHz, acetone-$d_6$) of **2**.

**Figure S12**. HMQC spectrum (500 MHz, acetone-$d_6$) of **2**.

**Figure S13**. HMBC spectrum (500 MHz, acetone-$d_6$) of **2**.

**Figure S14**. $^{1}$H-$^{1}$H COSY spectrum (500 MHz, acetone-$d_6$) of **2**.

**Figure S15**. NOESY spectrum (500 MHz, acetone-$d_6$) of **2**.

**Figure S1.** (**A**) Colony morphology of *Streptomyces* sp. OC1610.4. (**B**) 16S rRNA gene sequence of *Streptomyces* sp. OC1610.4 (GenBank number: MK045847). (**C**) Neighbor-joining phylogenetic tree based on 16S rRNA gene sequences of *Streptomyces* sp. OC1610.4.

**A.**

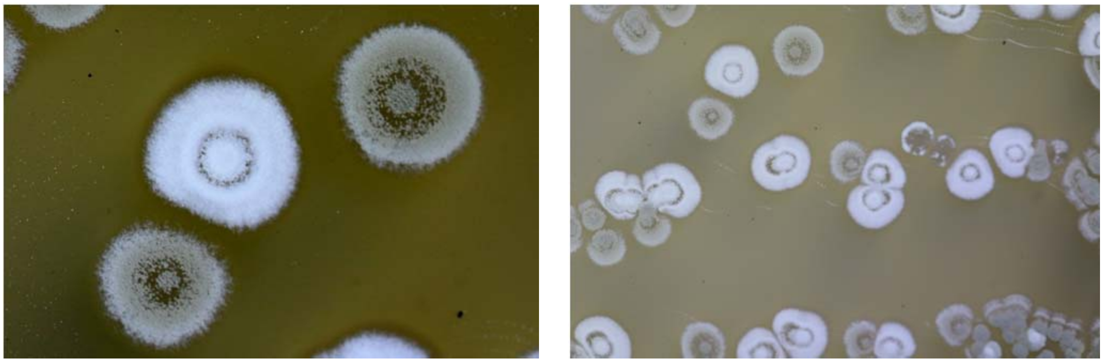

**B.**

GTGCAGTCGGGTCTTACCATGCAGTCGACGATGAAGCCCTTCGGGGTGGATTAGTGG
CGAACGGGTGAGTAACACGTGGGCAATCTGCCCTGCACTCTGGGACAAGCCCTGGAA
ACGGGGTCTAATACCGGATACGAGCCTCCCGGGCATCTGGGAGGTTGGAAAGCTCCG
GCGGTGCAGGATGAGCCCGCGGCCTATCAGCTTGTTGGTGAGGTAACGGCTCACCAA
GGCGACGACGGGTAGCCGGCCTGAGAGGGCGACCGGCCACACTGGGACTGAGACAC
GGCCCAGACTCCTACGGGAGGCAGCAGTGGGGAATATTGCACAATGGGCGAAAGCCT
GATGCAGCGACGCCGCGTGAGGGATGACGGCCTTCGGGTTGTAAACCTCTTTCAGCA
GGGAAGAAGCGAAAGTGACGGTACCTGCAGAAGAAGCGCCGGCTAACTACGTGCCA
GCAGCCGCGGTAATACGTAGGGCGCAAGCGTTGTCCGGAATTATTGGGCGTAAAGAG
CTCGTAGGCGGCTTGTCGCGTCGATTGTGAAAGCCCGGGGCTTAACTCCGGGTCTGCA
GTCGATACGGGCAGGCTAGAGTGTGGTAGGGGAGATCGGAATTCCTGGTGTAGCGGT
GAAATGCGCAGATATCAGGAGGAACACCGGTGGCGAAGGCGGATCTCTGGGCCATTA
CTGACGCTGAGGAGCGAAAGCGTGGGGAGCGAACAGGATTAGATACCCTGGTAGTCC
ACGCCGTAAACGGTGGGCACTATGTGTTGGCGACATTCCACGTCGTCGGTGCCGCAG
CTAACGCATTAAGAGCCCCGCCTGGGGGAGTACGGCCGCAACGCTGATACTCAAAAG
AATTGATGGGGGCGCGCACAAGCAGCGGAGCATGTGGCTTATTTCGACGCAACGCGC
AGAACTTACCGAGGCTTGACATACACCAGTAACATCCAGACATGAGTGCCCCCCTCC
TGGTCGGGGTACAGTAGTGCATGGGCTTGTCGTCAACTCAGTCGTGACATGTTGAGG
AGAAACCCCCCAGCACATCAGA

**C.**

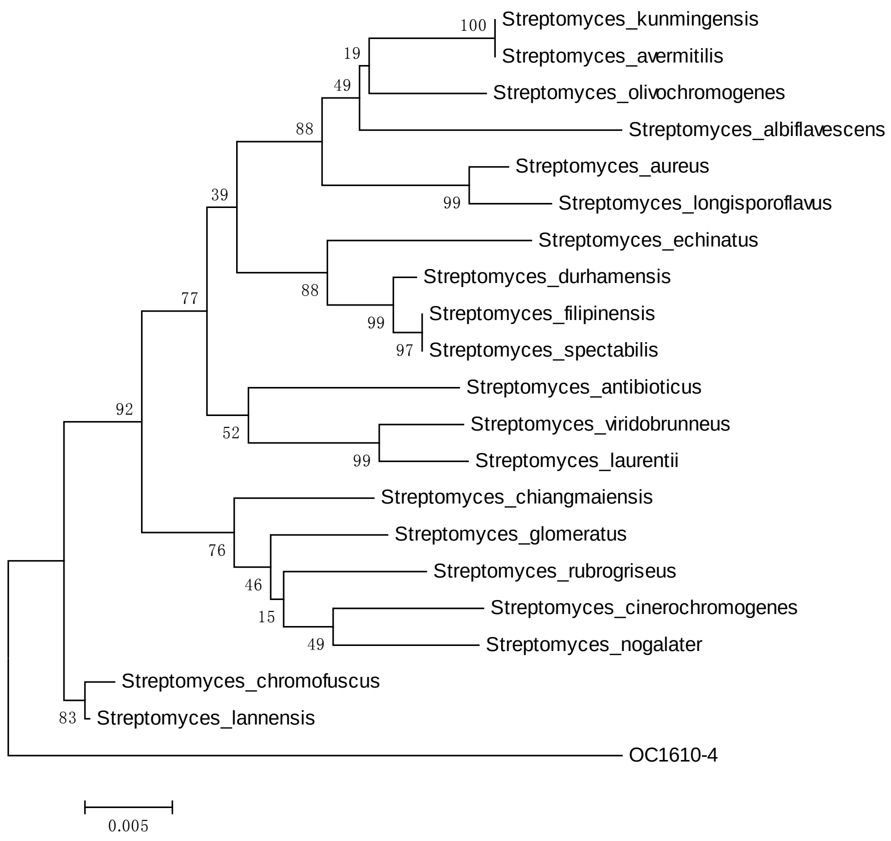


**Figure S2**. HR-ESI-MS of **1**.

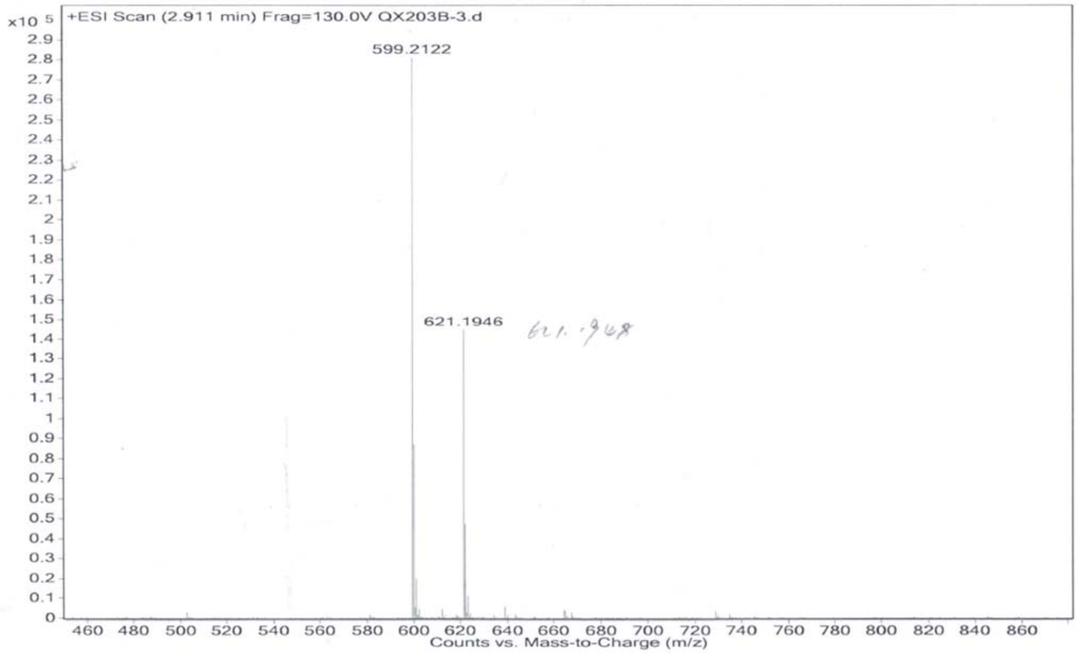

**Figure S3**. $^{1}$H-NMR spectrum (500 MHz, $CD_3OD$) of **1**.

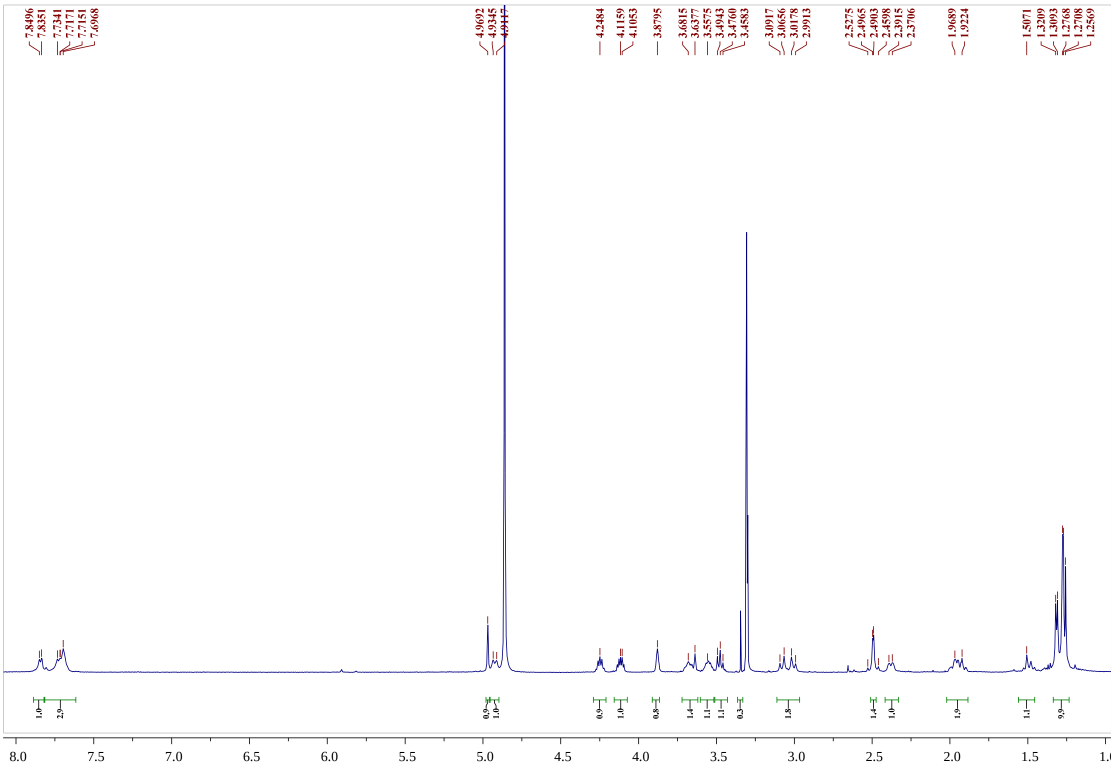


**Figure S4**. $^{13}$C-NMR spectrum (125 MHz, $CD_3OD$) of **1**.

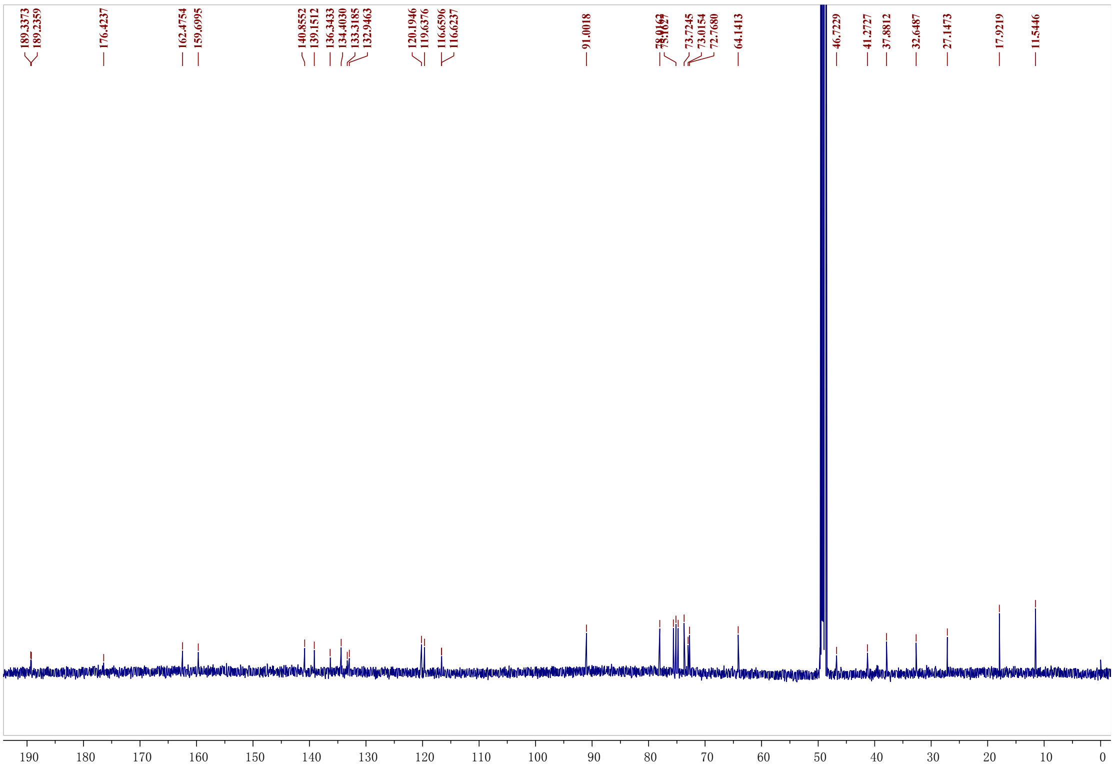

**Figure S5**. HMQC spectrum (500 MHz, $CD_3OD$) of **1**.

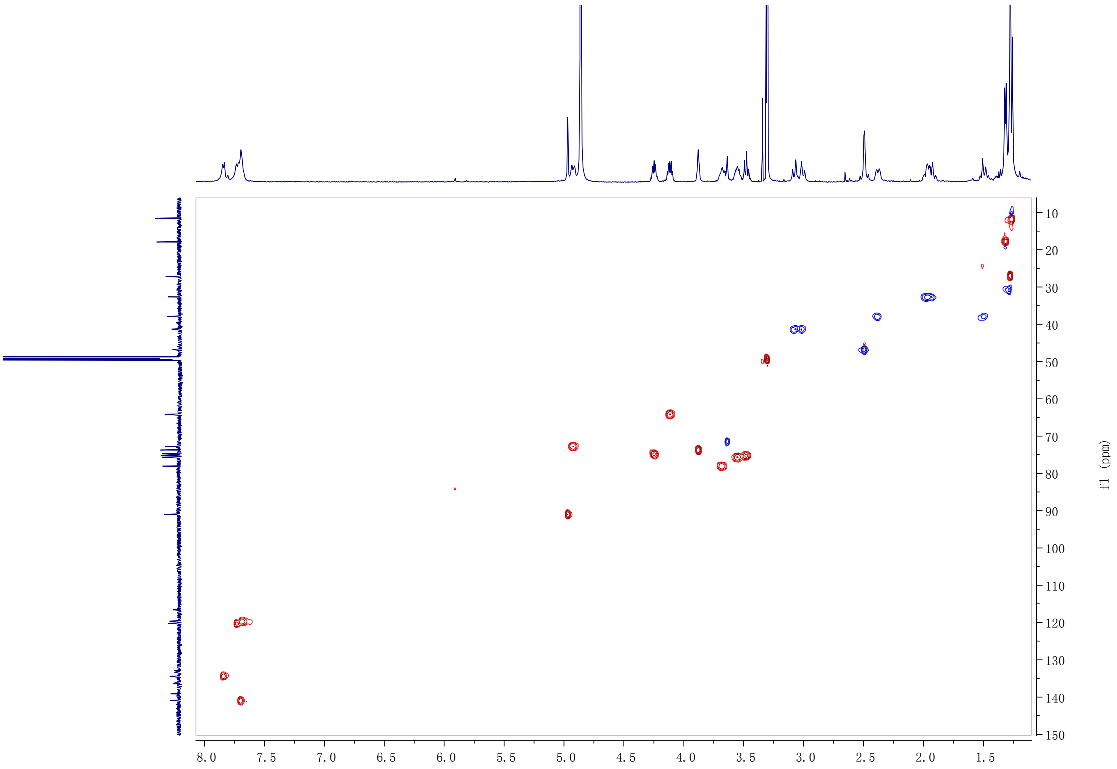


**Figure S6**. HMBC spectrum (500 MHz, $CD_3OD$) of **1**.

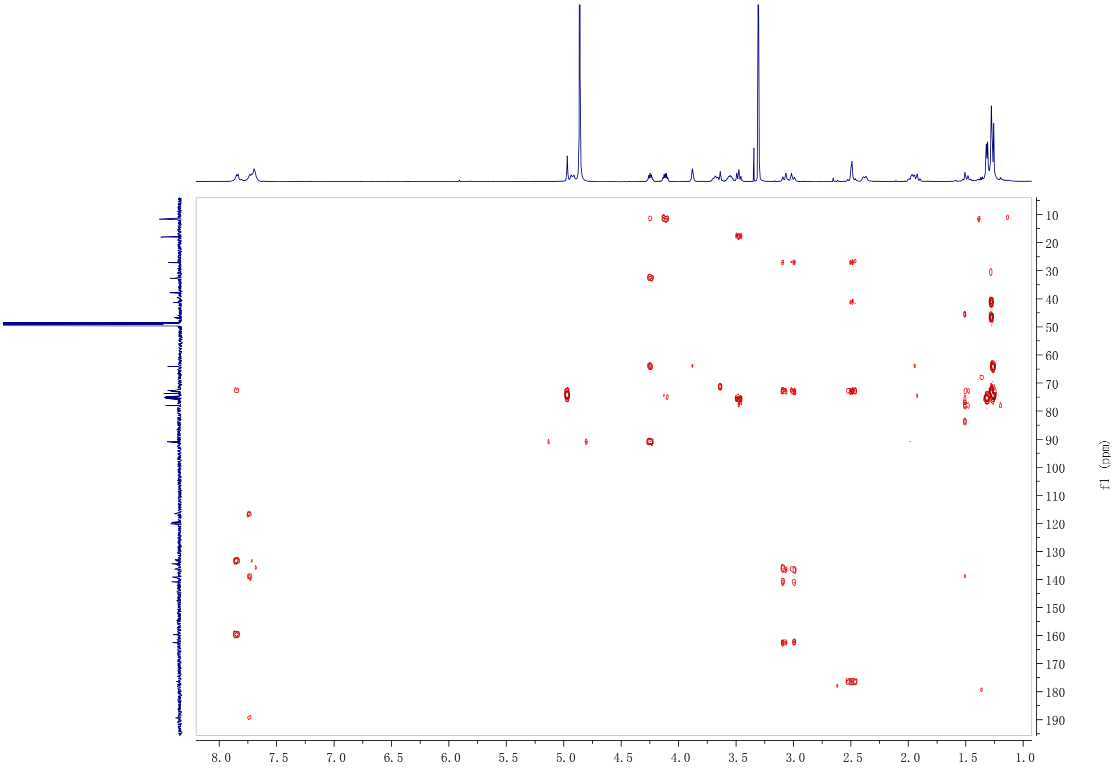

**Figure S7**. $^{1}$H-$^{1}$H COSY spectrum (500 MHz, $CD_3OD$) of **1**.

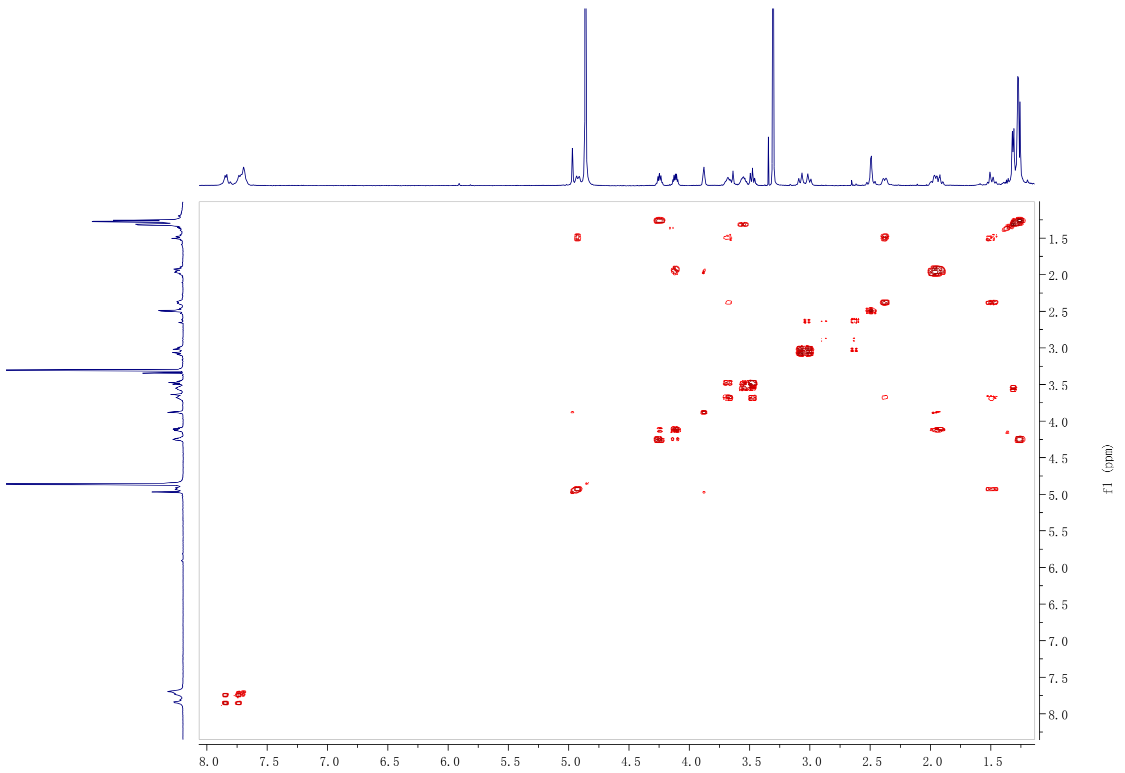


**Figure S8**. NOESY spectrum (500 MHz, $CD_3OD$) of **1**.

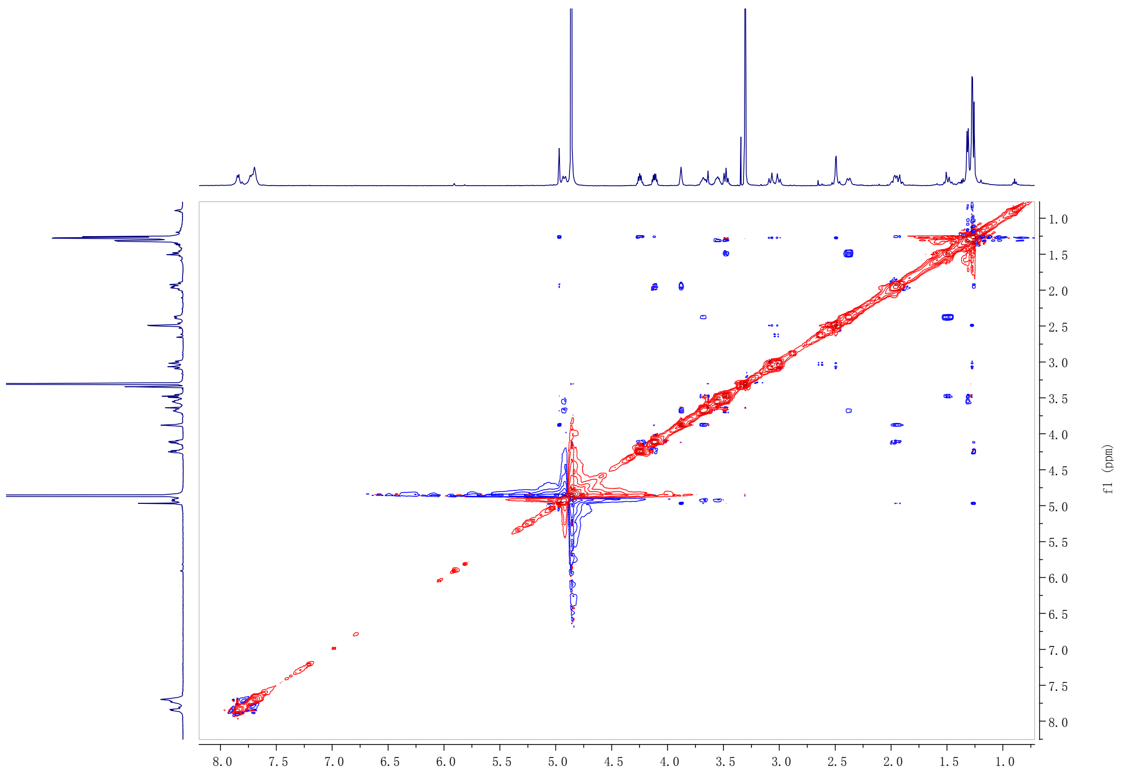

**Figure S9**. HR-ESI-MS of **2**.

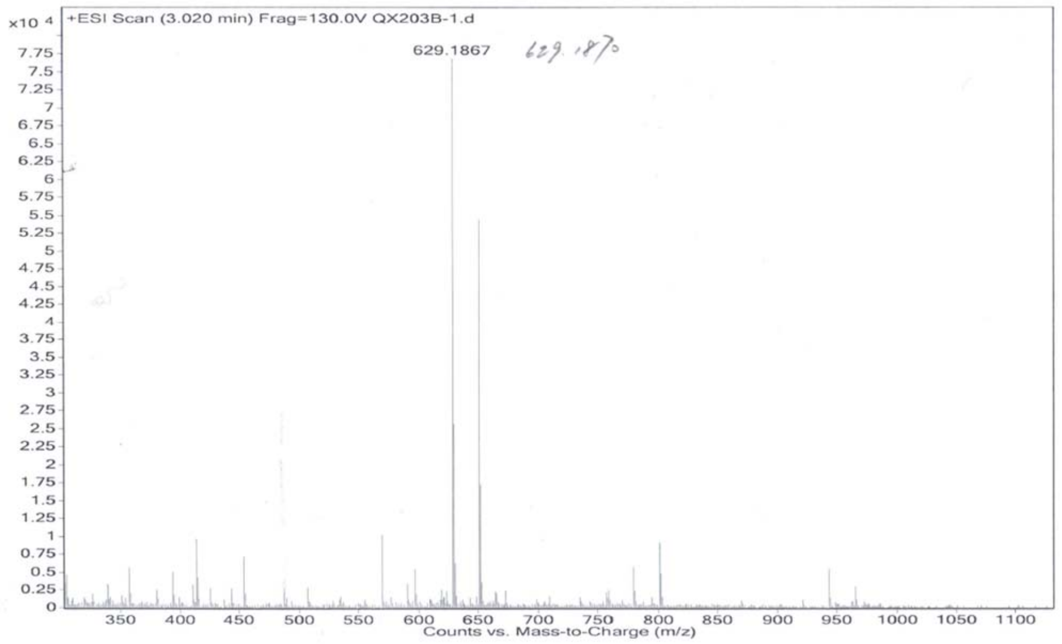


**Figure S10**. $^{1}$H-NMR spectrum (500 MHz, acetone-$d_6$) of **2**.

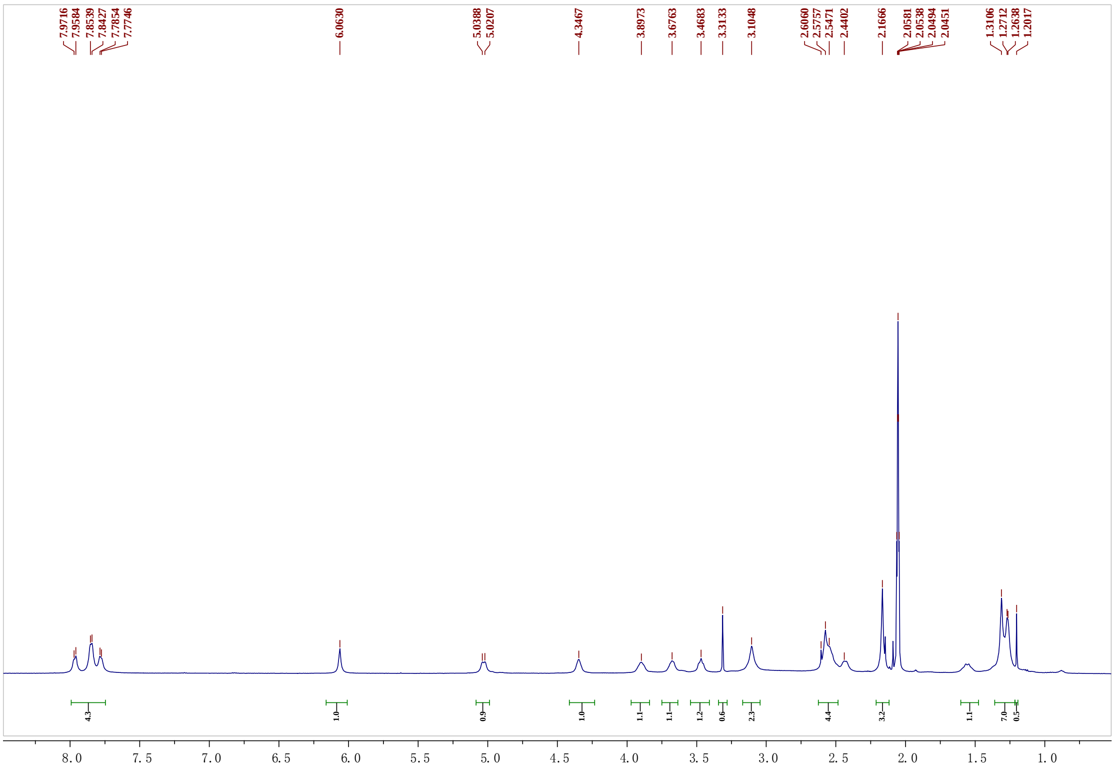

**Figure S11**. $^{13}$C-NMR spectrum (125 MHz, acetone-d$_6$) of **2**.

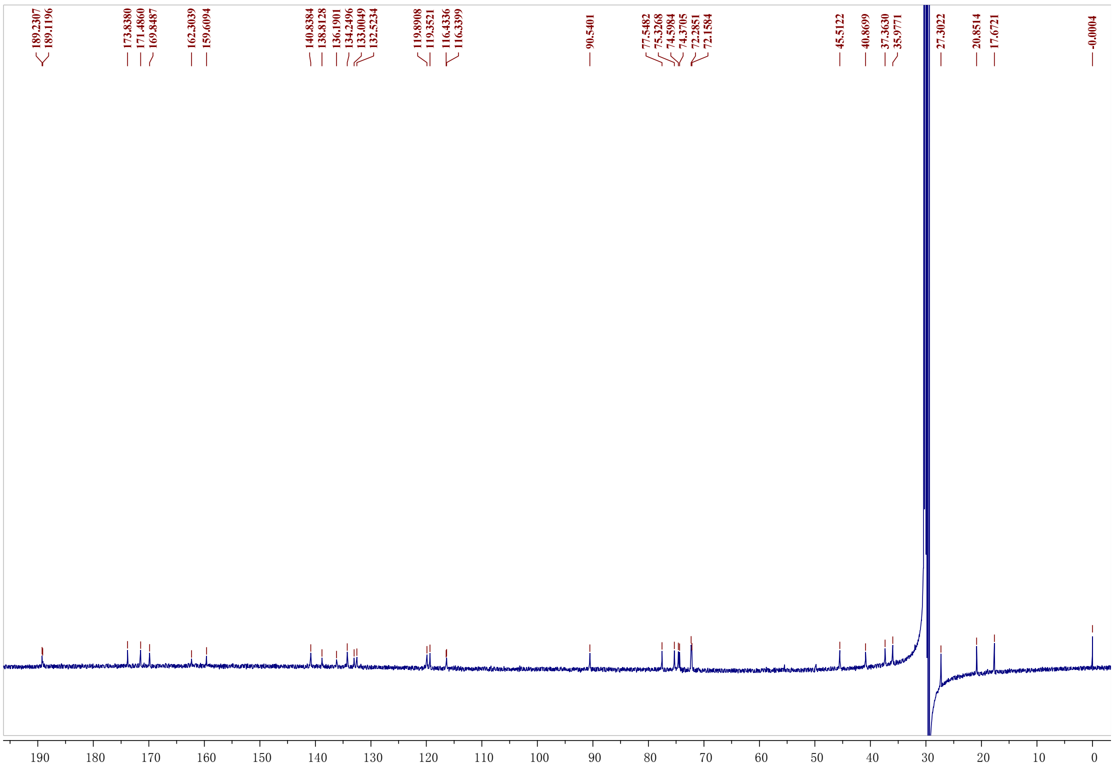


**Figure S12**. HMQC spectrum (500 MHz, acetone-d$_6$) of **2**.

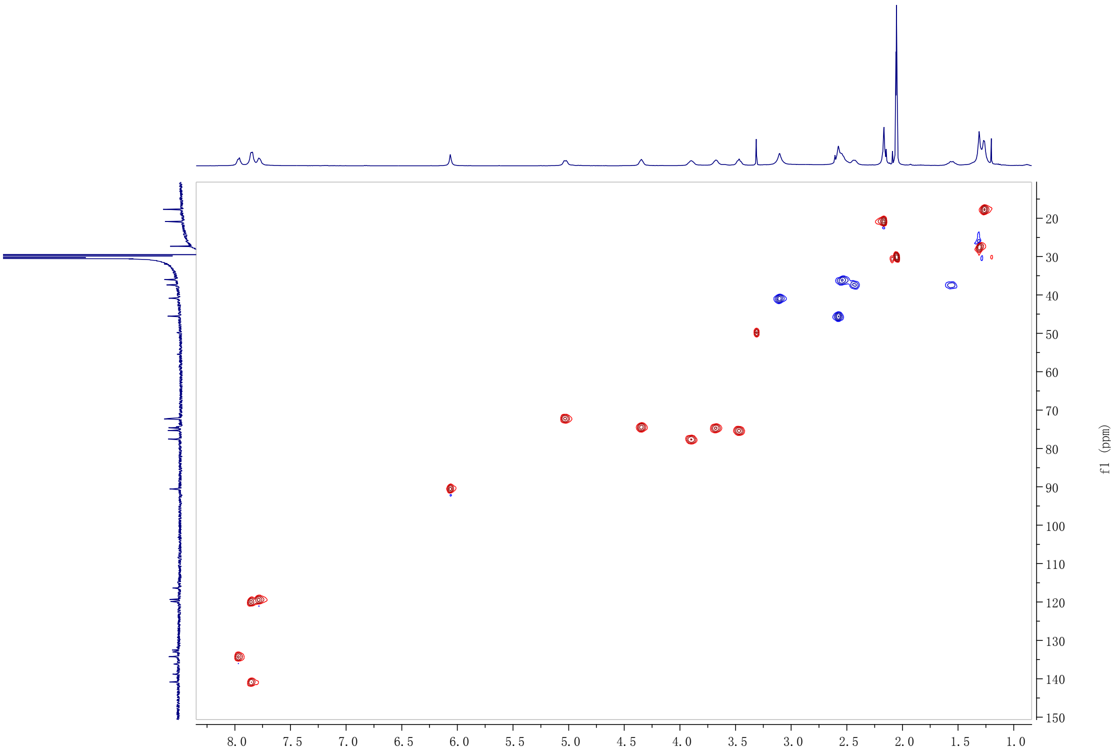

**Figure S13**. HMBC spectrum (500 MHz, acetone-$d_6$) of **2**.

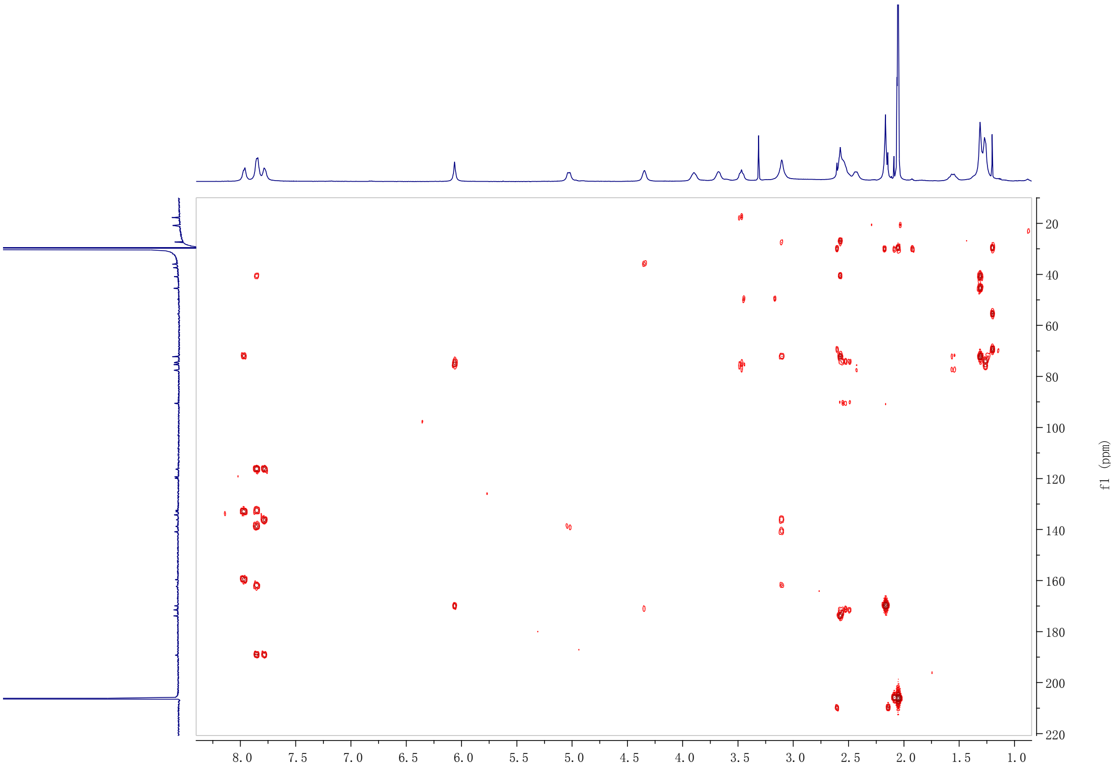


**Figure S14**. $^{1}$H-$^{1}$H COSY spectrum (500 MHz, acetone-$d_6$) of **2**.

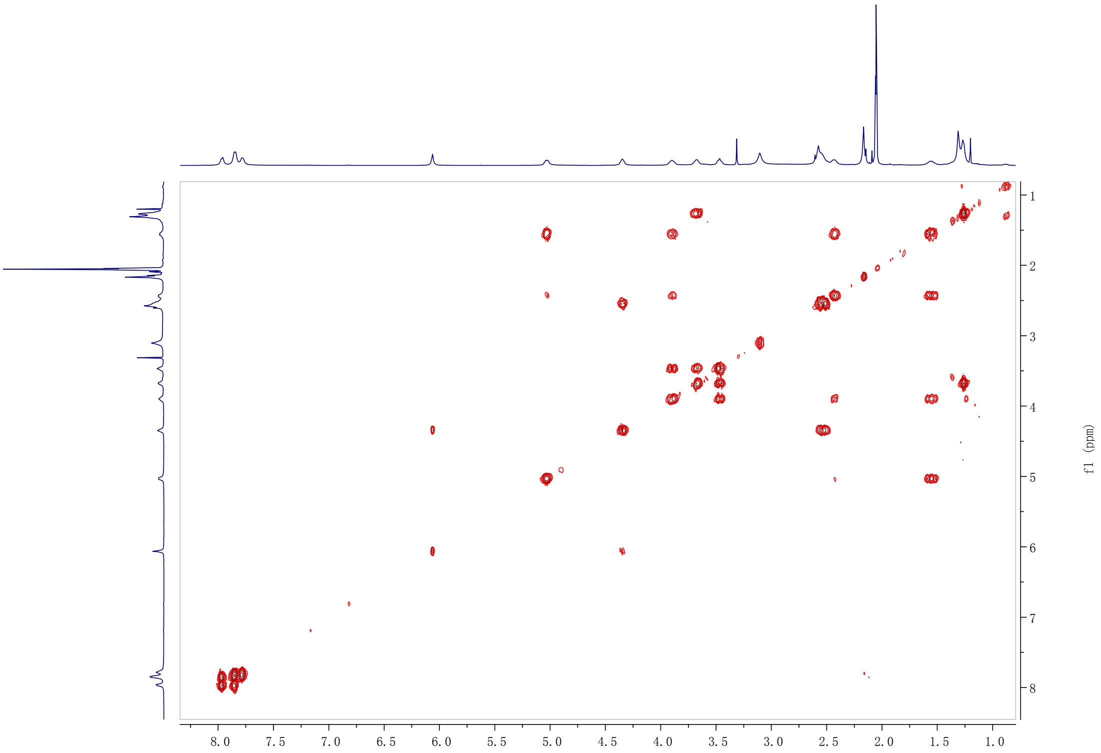

**Figure S15**. NOESY spectrum (500 MHz, acetone-$d_6$) of **2**.

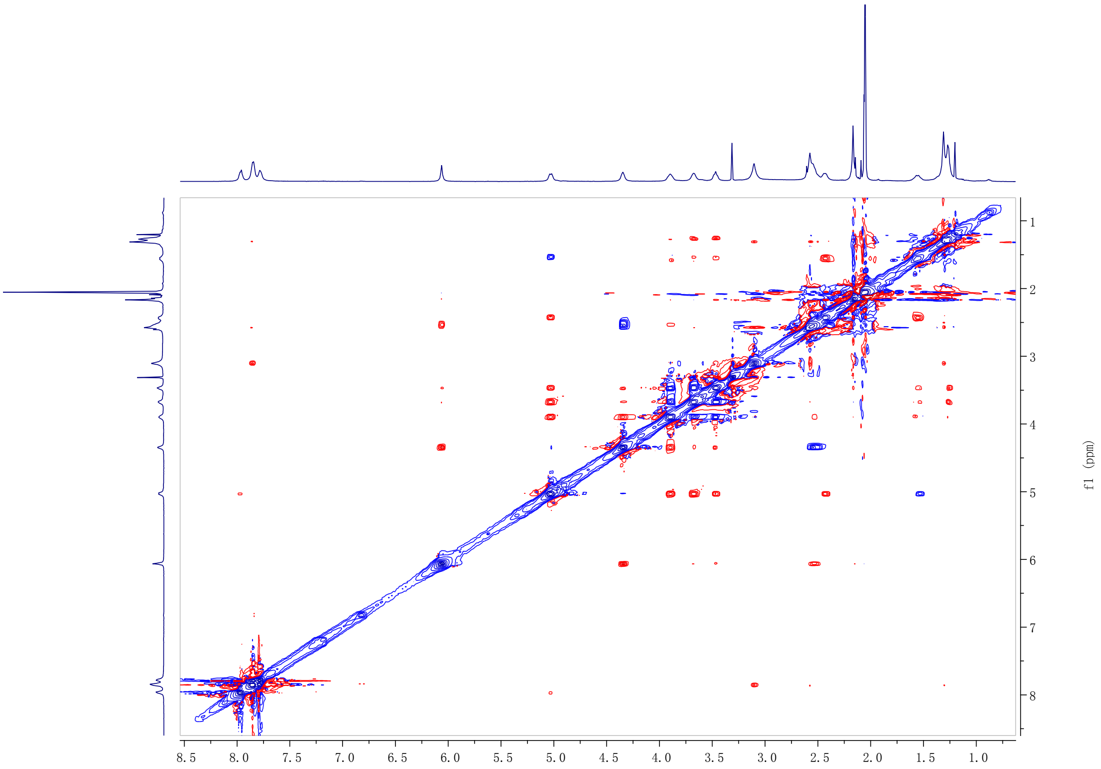

Supplement: Supplementary file 1 [file marinedrugs-17-00277-s001.pdf]
